# Supplementary material for: Dysregulation of Locus-Specific Repetitive Elements in TCGA Pan-Cancers
Source: Genes (Basel). 2025 Apr 29;16(5):528. doi: 10.3390/genes16050528 (PMC12111754; doi:10.3390/genes16050528)
Supplement: Supplementary file 1 [file genes-16-00528-s001.zip › TCGA_repeats_supplementary_figures_revised_tracked.pdf]

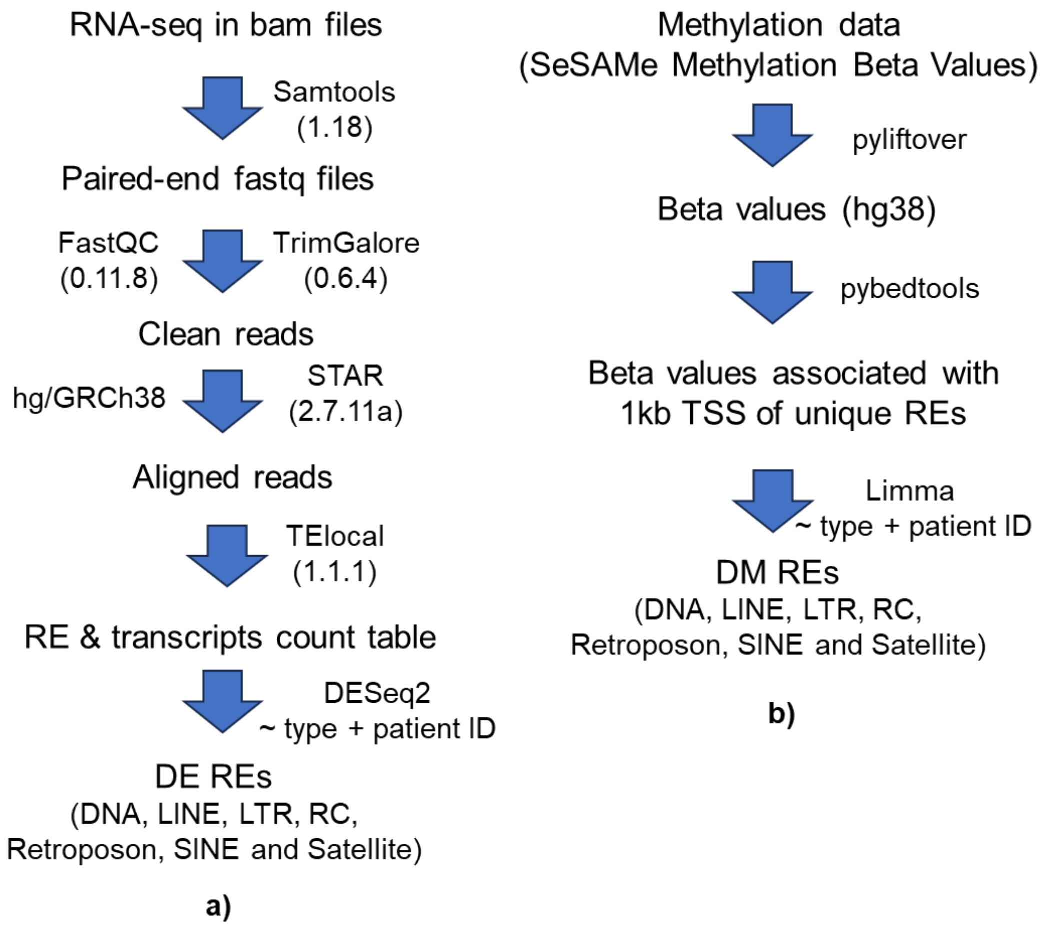

Figure S1: The workflow to identify dysregulated REs across 12 cancer types. **a):** The workflow for RE expression analysis; **b):** The workflow for RE methylation analysis.

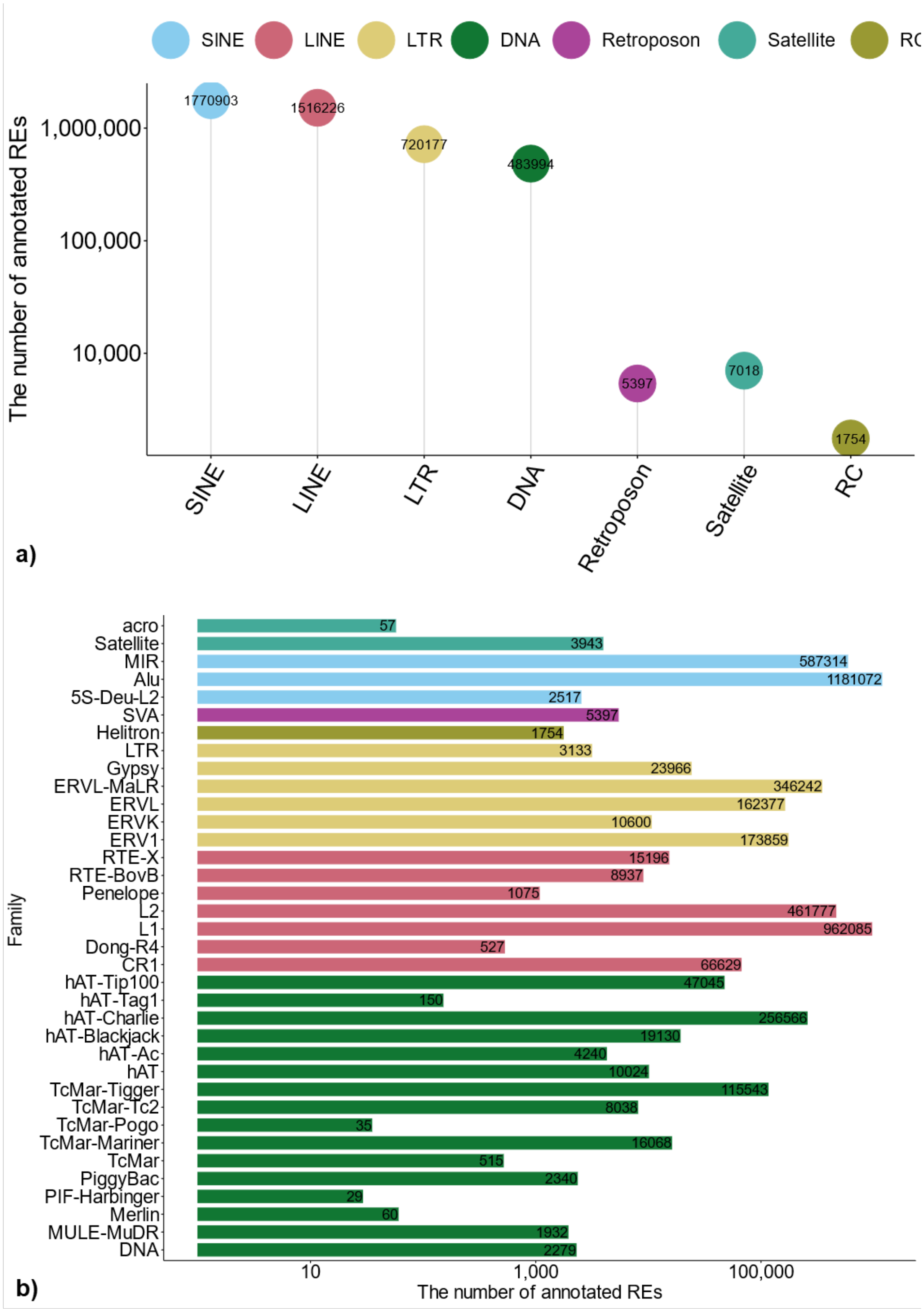

Figure S2: Summary of locus-specific REs based on hg38 annotation for TElocal. a): Number of locus-specific RE elements for each of 7 RE classes; **b):** Number of locus-specific RE elements in each RE family.

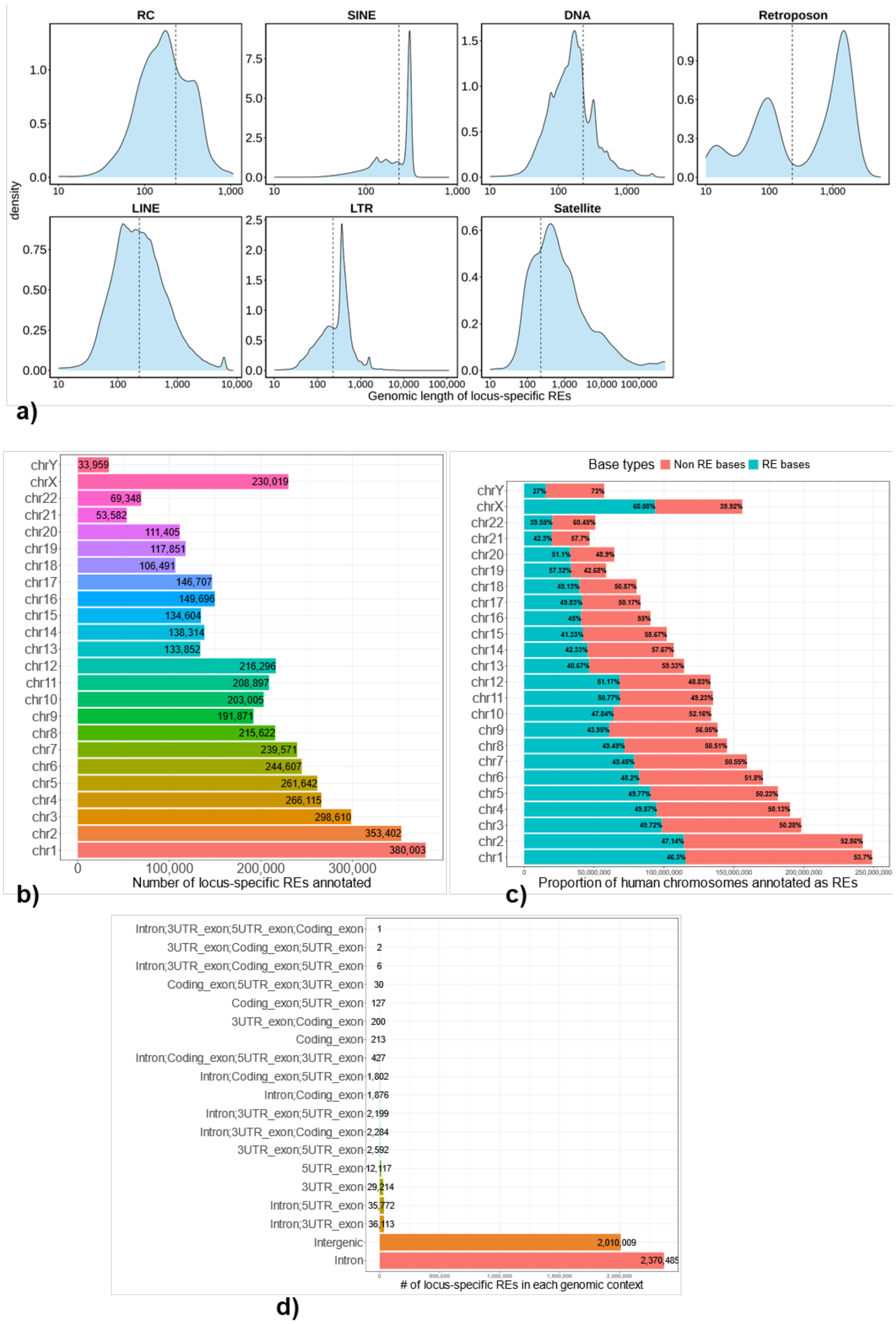

Figure S3: Distribution of locus-specific REs in the human genome based on hg38 annotation for TElocal. a): genomic length of each RE class in the human genome; **b):** number of locus-specific REs in each chromosome; **c):** percentage of nucleotide bases in each chromosome that are annotated as REs; **d):** number of locus-specific REs with different genomic contexts based on protein-coding genes.

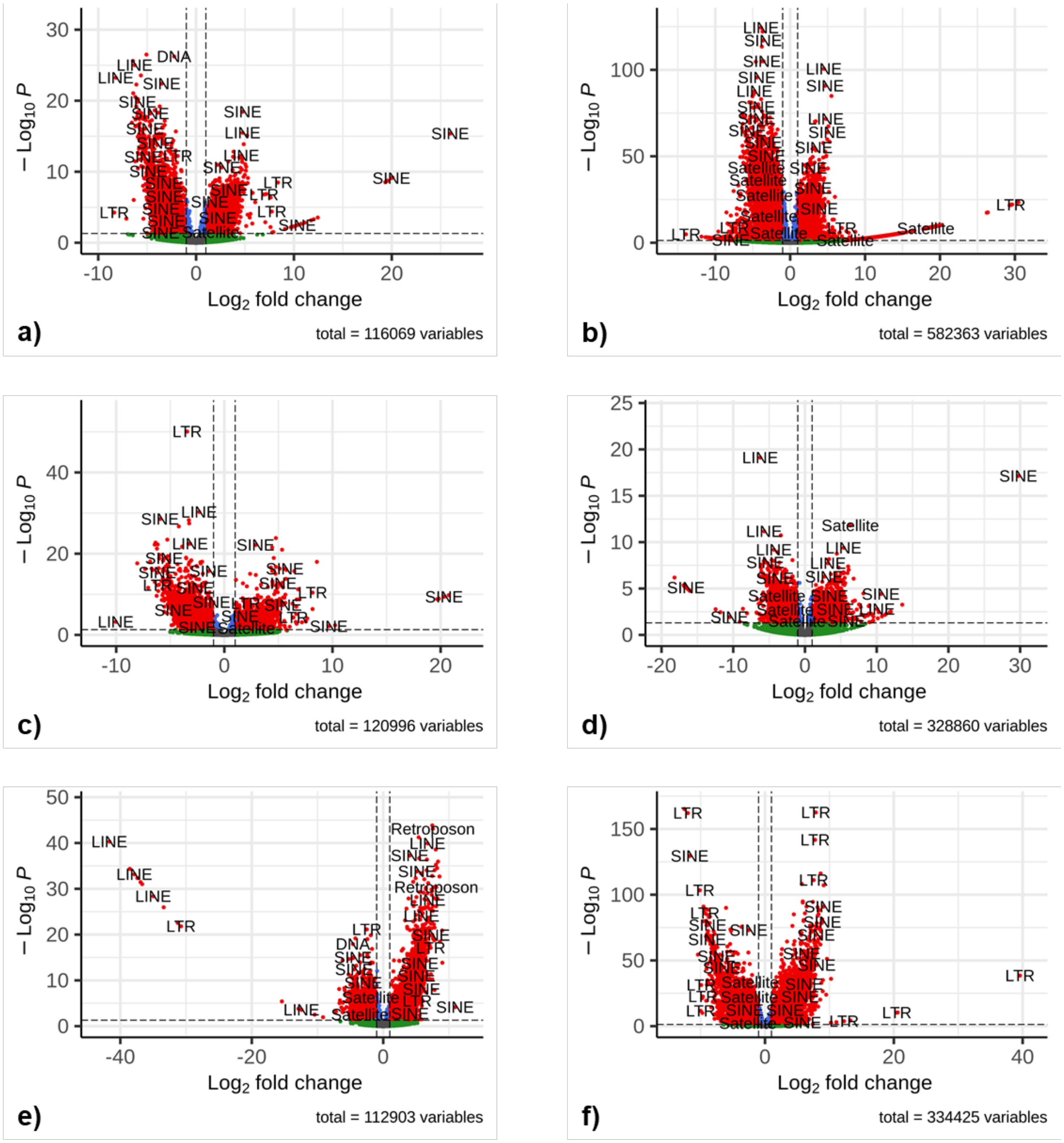

a)
− Log10 P
Log2 fold change
total = 116069 variables
b)
− Log10 P
Log2 fold change
total = 582363 variables
c)
− Log10 P
Log2 fold change
total = 120996 variables
d)
− Log10 P
Log2 fold change
total = 328860 variables
e)
− Log10 P
Log2 fold change
total = 112903 variables
f)
− Log10 P
Log2 fold change
total = 334425 variables

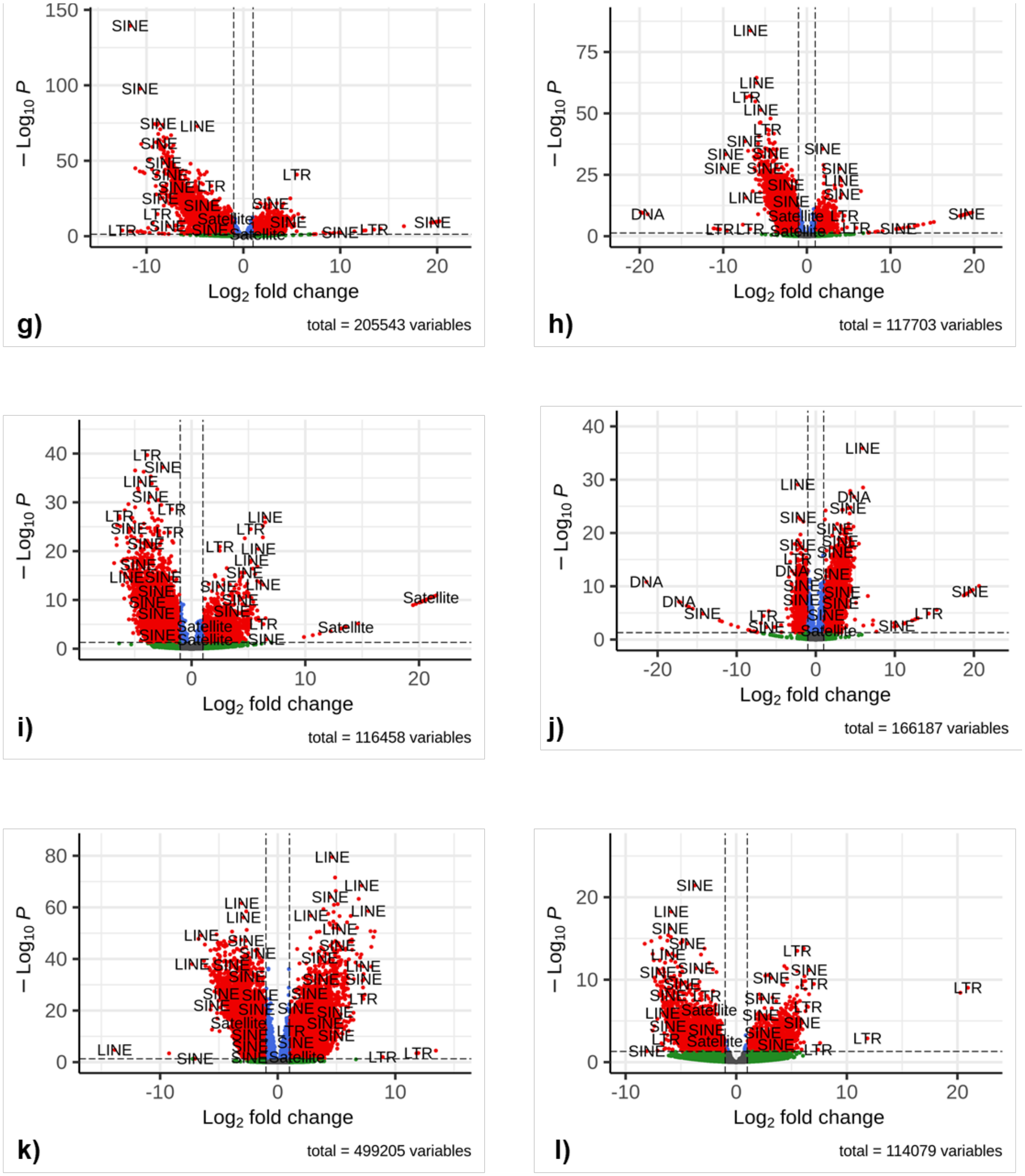

Figure S4: Expression changes of locus-specific REs in each of the 12 cancer types (REs with |log2 fold change| >= 1 and adjusted p-values <= 0.05 are considered differentially expressed REs and represented by red points)**. a):** expression changes of locus-specific REs for BLCA between tumors and matched normal samples; **b):** for BRCA; **c):** for COAD; **d):** for ESCA; **e):** for HNSC; **f):** for KIRC; **g):** for KIRP; **h):** for LIHC; **i):** for LUAD; **j):** for PRAD; **k):** for THCA; **l):** for UCEC;

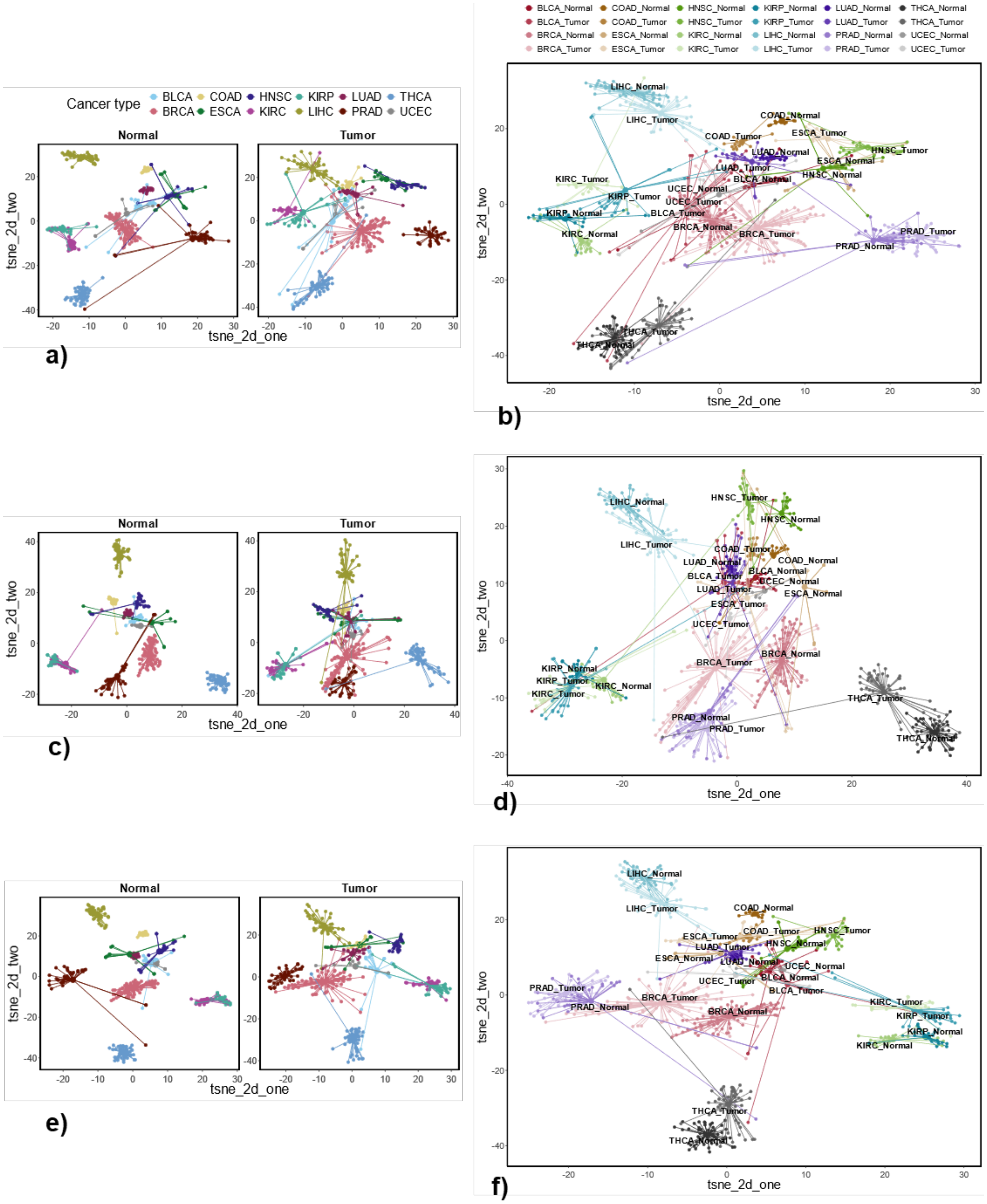

Figure S5. Sample clustering with uniquely dysregulated intergenic REs identified across 12 cancer types. a): t-SNE plots based on uniquely up-regulated intergenic REs for Normal and Tumor sample clustering separately; **b):** t-SNE plot based on uniquely up-regulated intergenic

REs for different sample type clustering (tumor with matched normal samples); **c):** t-SNE plots based on uniquely down-regulated intergenic REs for Normal and Tumor sample clustering separately; **d):** t-SNE plot based on uniquely down-regulated intergenic REs for different sample type clustering (tumor with matched normal samples); **e):** t-SNE plots based on uniquely up- and down-regulated intergenic REs for Normal and Tumor sample clustering separately; **f):** t-SNE plot based on uniquely up- and down-regulated intergenic REs for different sample type clustering (tumor with matched normal samples);

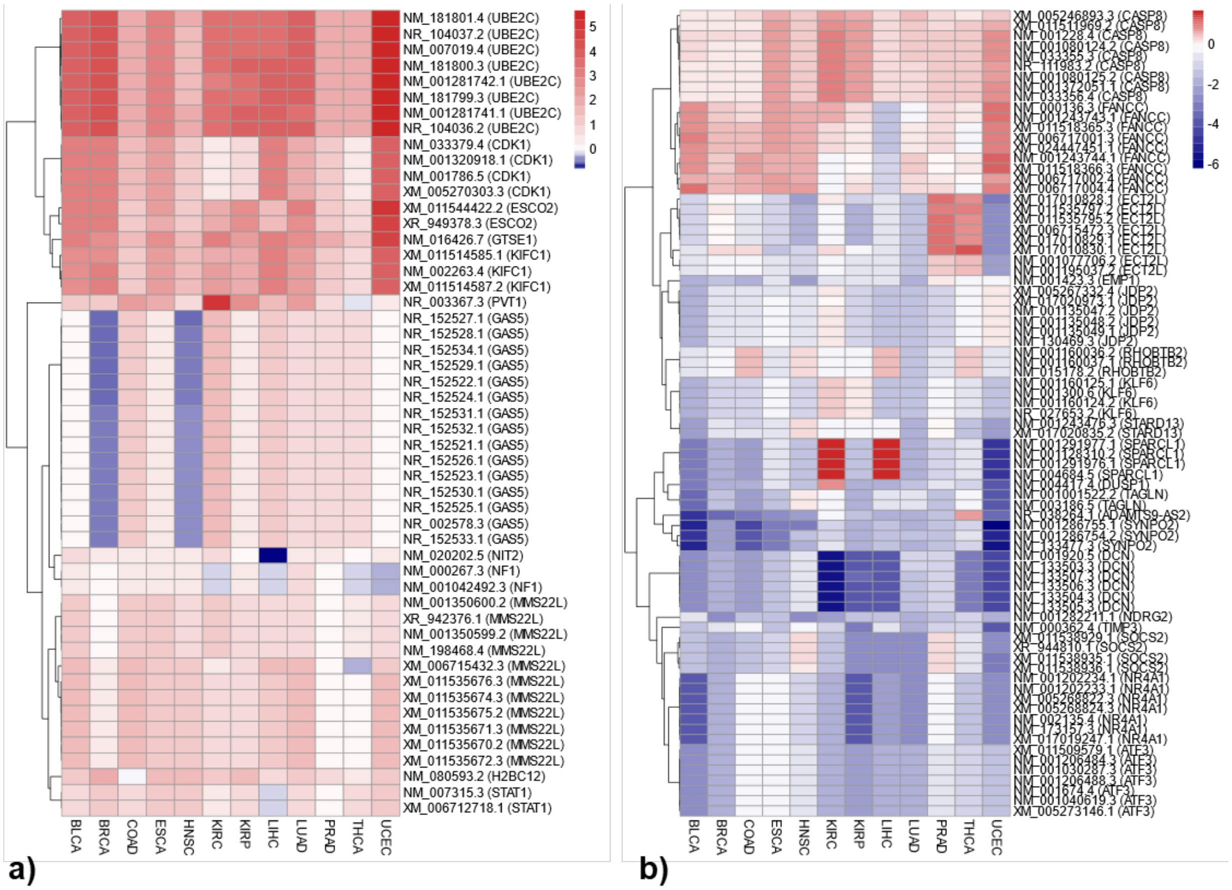

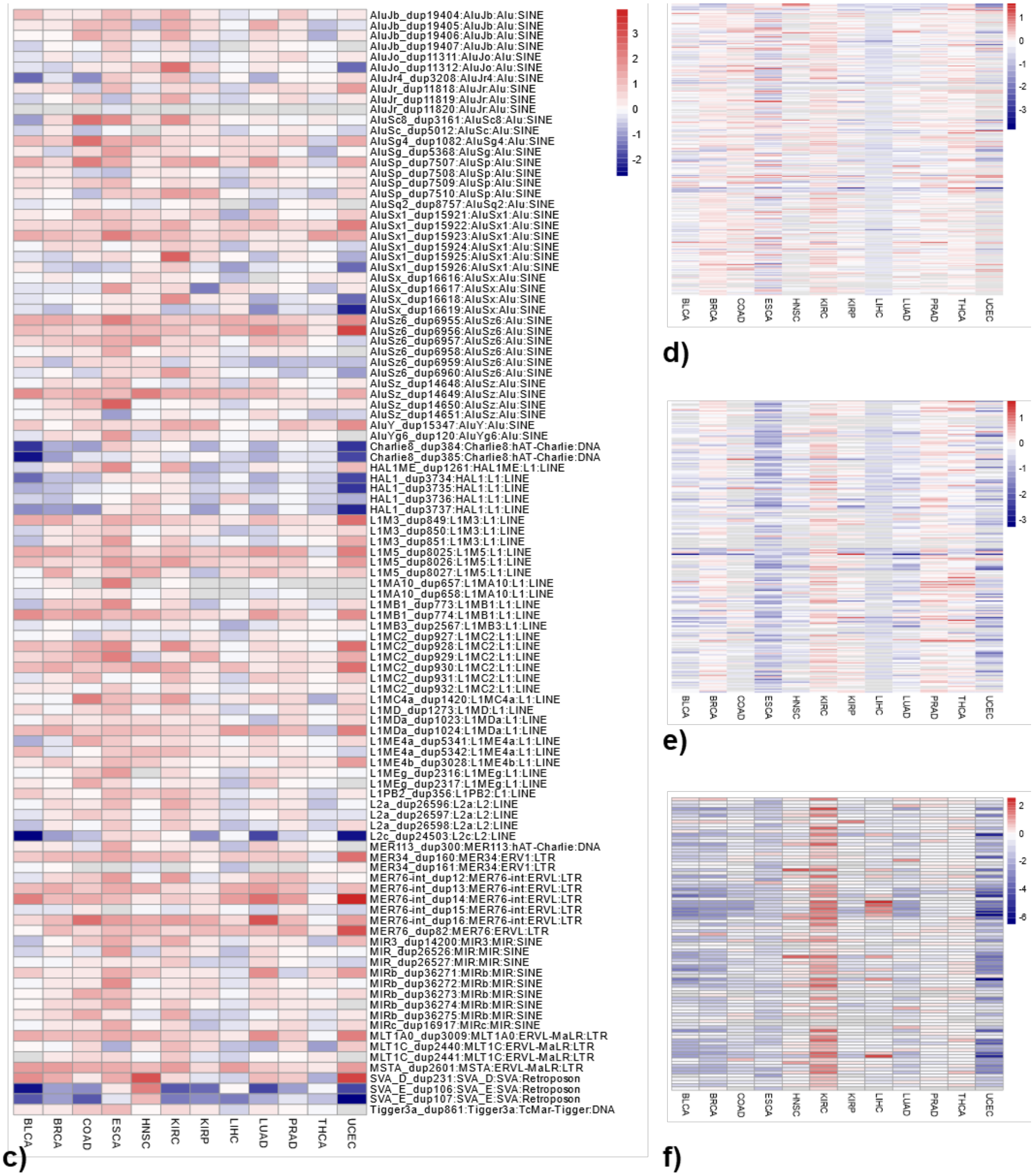

Figure S6: Expression changes of transcripts corresponding to cancer genes associated with recurrently dysregulated genic REs. a): expression changes of transcripts (that are cancer genes) associated with recurrently up-regulated genic REs; **b):** expression changes of transcripts (that are cancer genes) associated with recurrently down-regulated genic REs; **c):** expression changes of REs that are associated with gene CASP8; **d):** expression changes of REs that are associated with gene FANCC; **e):** expression changes of REs that are associated with gene ECT2L; **f):** expression changes of REs that are associated with gene SPARCL1;

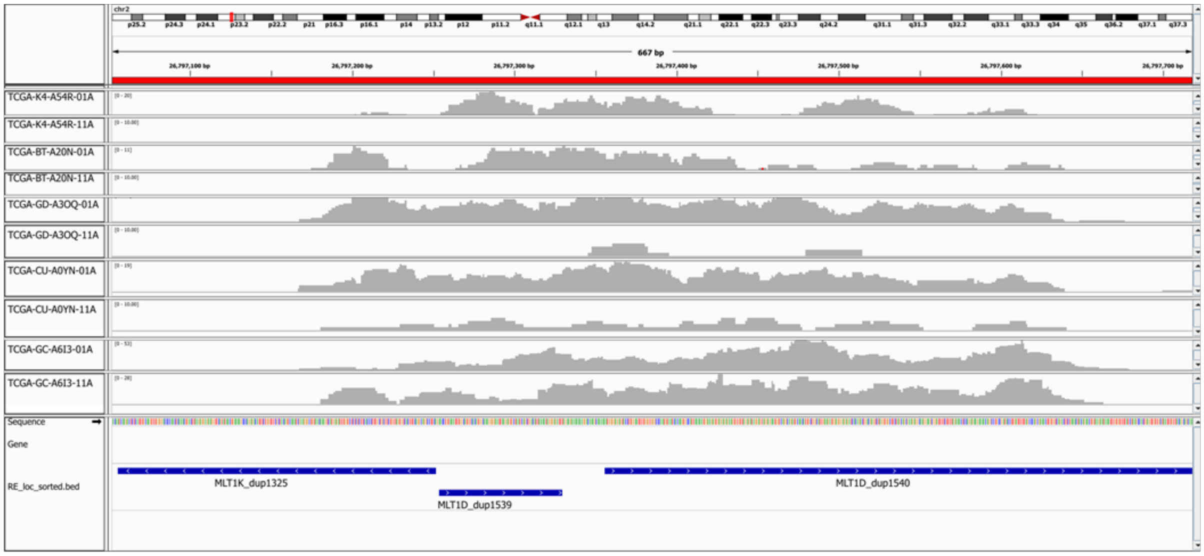

a)

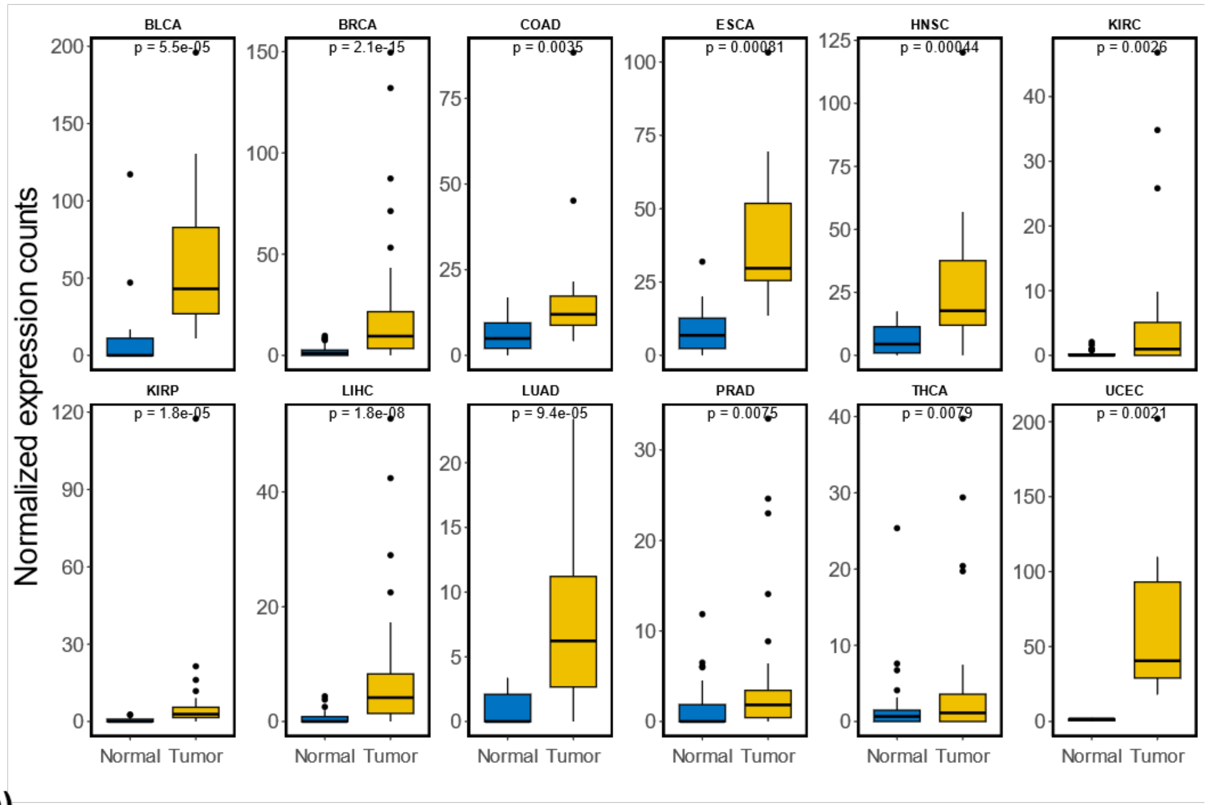

b)

Figure S7: Consistent up-regulated intergenic RE (*i.e.*, MLT1D_dup1540) **across 12 cancer types. a):** reads coverage corresponding to MLT1D_dup1540 between the randomly selected 5 tumor and matched normal samples in BLCA (tumor sample ends with 01A while the matched normal sample ends with 11A); **b):** normalized expression comparison between tumor and matched normal samples for MLT1D_dup1540 across 12 cancer types.

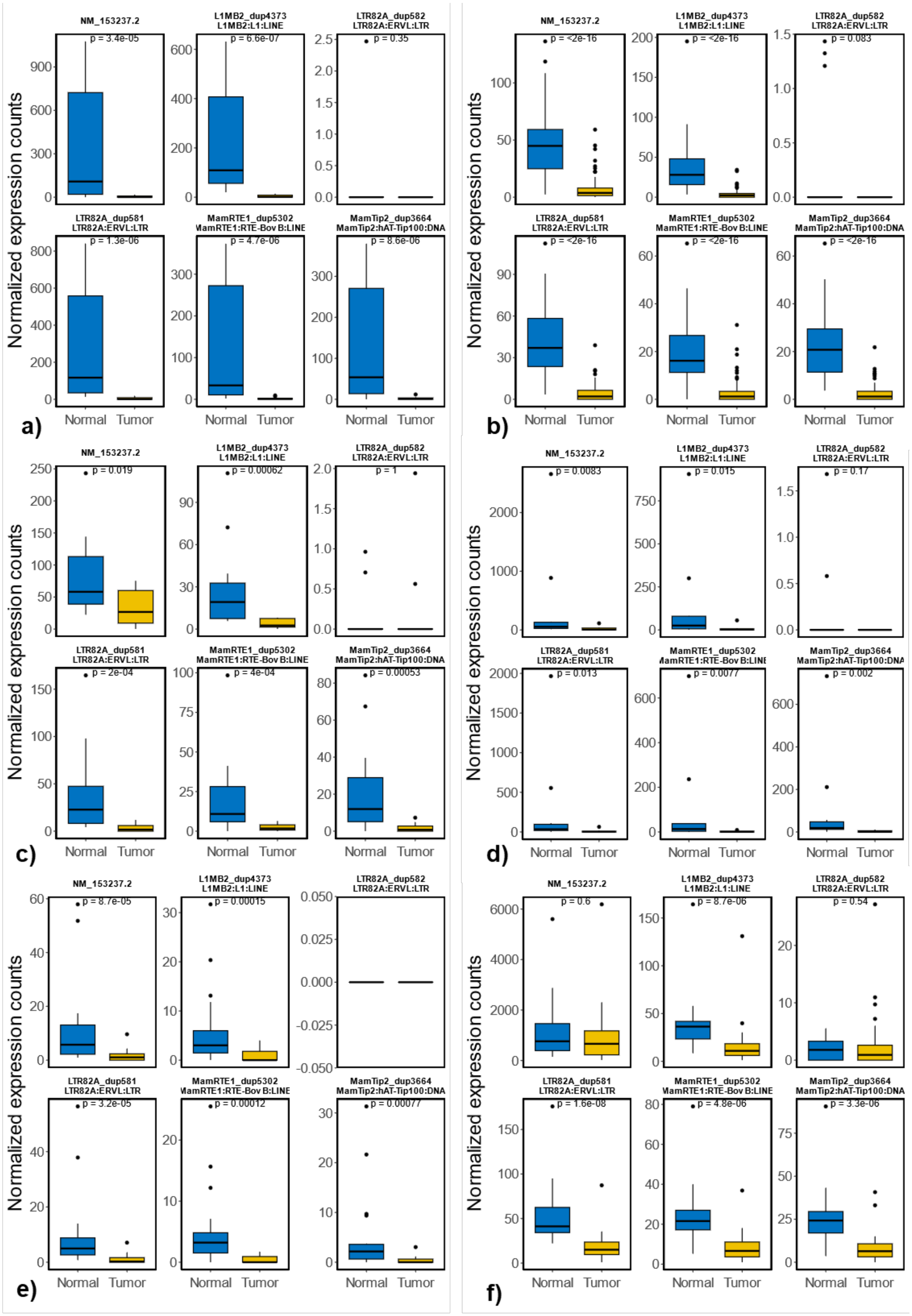
a)
b)
c)
d)
e)
f)
Normalized expression counts
Normal
Tumor
NM_153237.2
L1MB2_dup4373
L1MB2:L1:LINE
LTR82A_dup582
LTR82A:ERVL:LTR
LTR82A_dup581
LTR82A:ERVL:LTR
MamRTE1_dup5302
MamRTE1:RTE-BovB:LINE
MamTip2_dup3664
MamTip2:hAT-Tip100:DNA
p = 3.4e-05
p = 6.6e-07
p = 0.35
p = 1.3e-06
p = 4.7e-06
p = 8.6e-06
p = <2e-16
p = 0.083
p = 0.019
p = 0.00062
p = 1
p = 2e-04
p = 4e-04
p = 0.00053
p = 0.0083
p = 0.015
p = 0.17
p = 0.013
p = 0.0077
p = 0.002
p = 8.7e-05
p = 0.00015
p = 3.2e-05
p = 0.00012
p = 0.00077
p = 0.6
p = 8.7e-06
p = 0.54
p = 1.6e-08
p = 4.8e-06
p = 3.3e-06

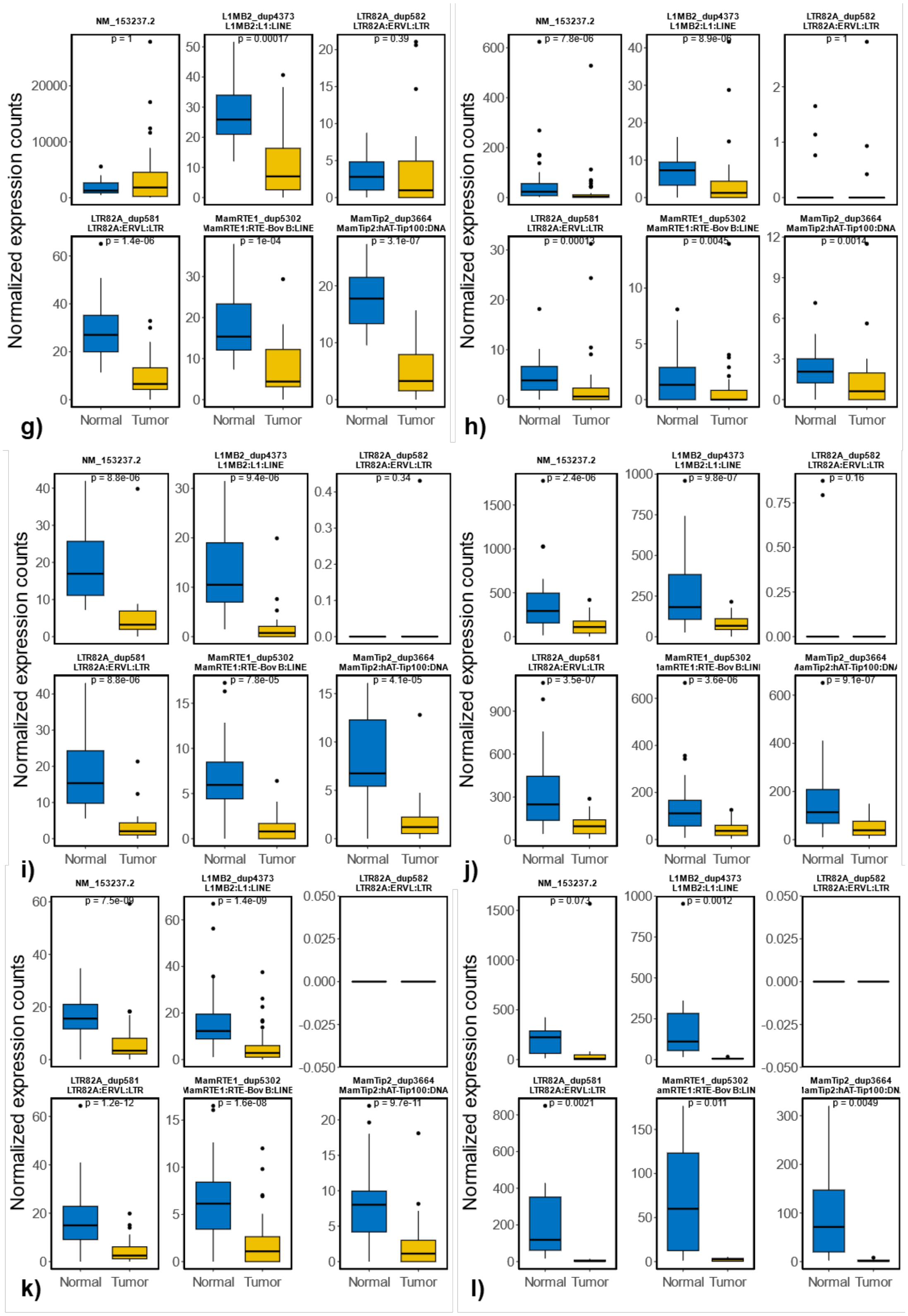

Figure S8: Expression comparison between tumor and matched normal samples for TMEM252 gene (with transcripts ID: NM_153237.2) and its associated REs across 12 cancer

types. a): for BLCA; **b):** for BRCA; **c):** for COAD; **d):** for ESCA; **e):** for HNSC; **f):** for KIRC; **g):** for KIRP; **h):** for LIHC; **i):** for LUAD; **j):** for PRAD; **k):** for THCA; **l):** for UCEC;

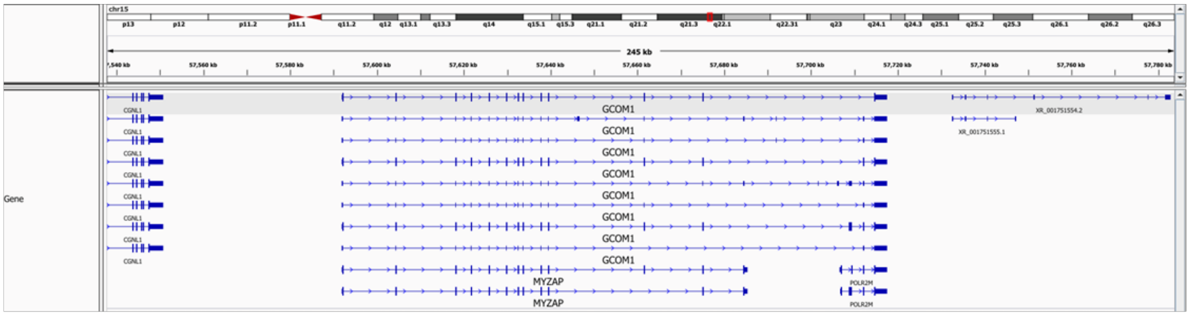

a)

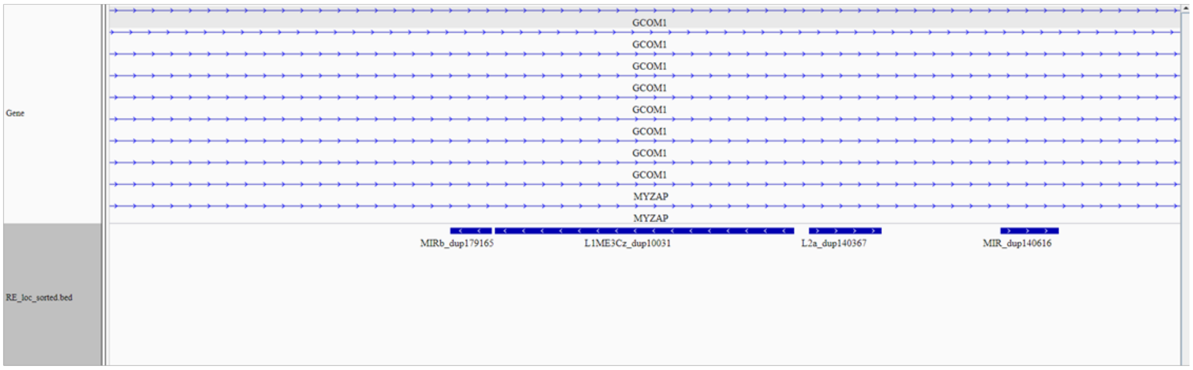

b)

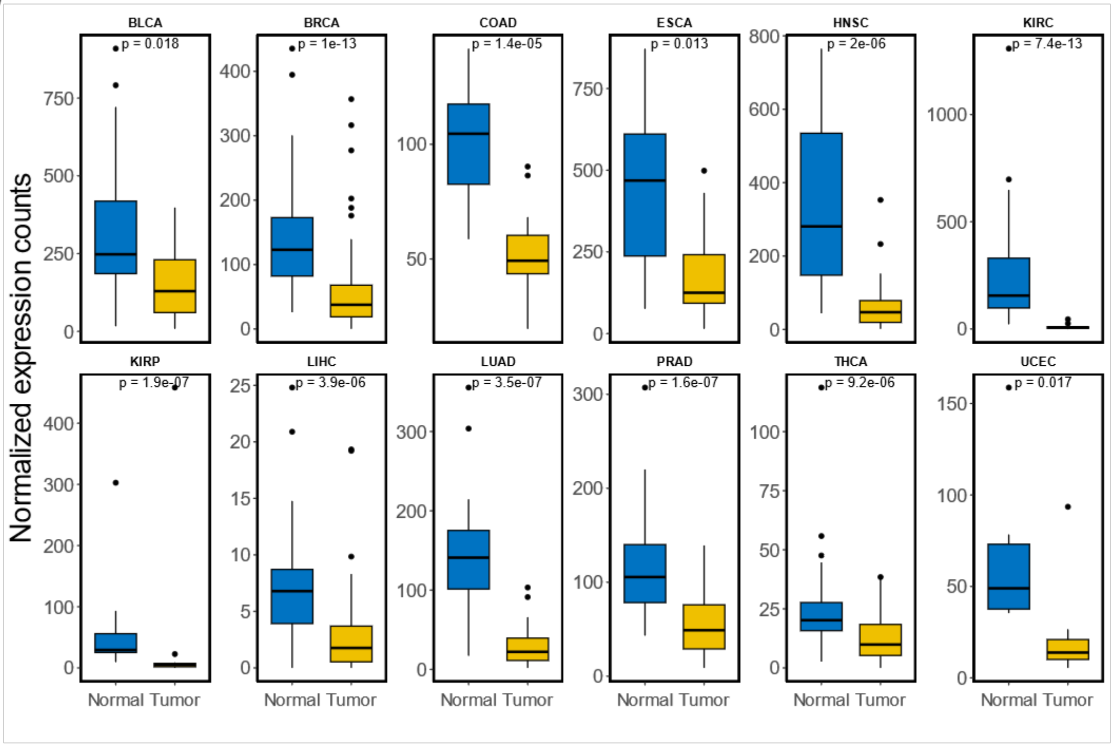

c)

Figure S9: Consistent down-regulated intronic RE (*i.e.*, L1ME3Cz_dup10031) **across 12 cancer types. a): annotation for** L1ME3Cz_dup10031 associated genes; **b):** genomic context

corresponding to L1ME3Cz_dup10031 in the human genome; **c):** normalized expression comparison between tumor and matched normal samples for L1ME3Cz_dup10031 across 12 cancer types.

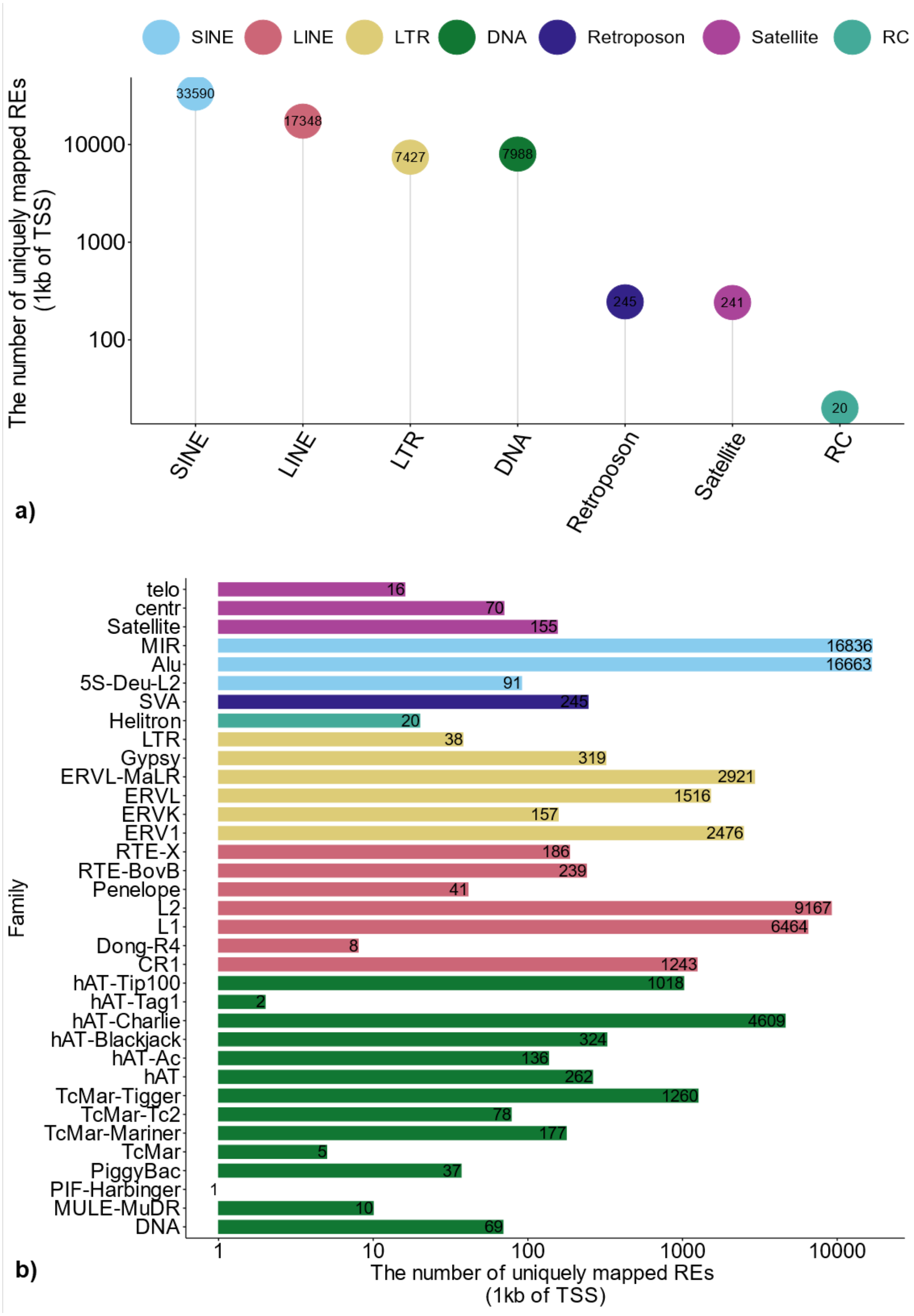
SINE
LINE
LTR
DNA
Retroposon
Satellite
RC
The number of uniquely mapped REs (1kb of TSS)
33590
17348
7427
7988
245
241
20
10000
1000
100
a)
Family
telo 16
centr 70
Satellite 155
MIR 16836
Alu 16663
5S-Deu-L2 91
SVA 245
Helitron 20
LTR 38
Gypsy 319
ERVL-MaLR 2921
ERVL 1516
ERVK 157
ERV1 2476
RTE-X 186
RTE-BovB 239
Penelope 41
L2 9167
L1 6464
Dong-R4 8
CR1 1243
hAT-Tip100 1018
hAT-Tag1 2
hAT-Charlie 4609
hAT-Blackjack 324
hAT-Ac 136
hAT 262
TcMar-Tigger 1260
TcMar-Tc2 78
TcMar-Mariner 177
TcMar 5
PiggyBac 37
PIF-Harbinger 1
MULE-MuDR 10
DNA 69
1
10
100
1000
10000
The number of uniquely mapped REs (1kb of TSS)
b)

Fig. S10: Number of REs that can be uniquely mapped with DNA methylation probes. a): Number of locus-specific RE elements for each of 7 RE classes that can be uniquely mapped with DNA methylation probes; **b):** Number of locus-specific RE elements in each RE family that can be uniquely mapped with DNA methylation probes.

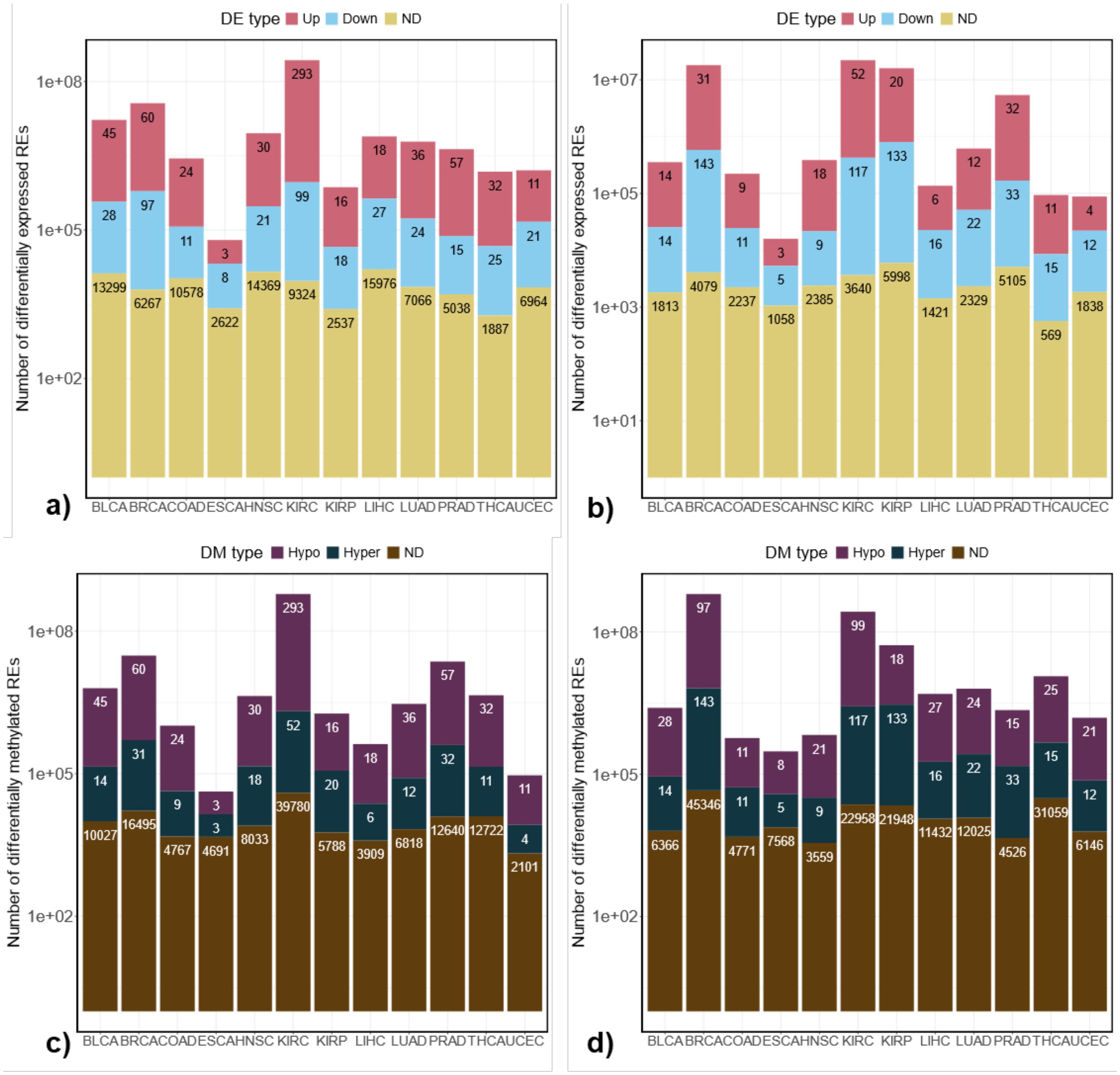

Fig. S11: Number of REs that are differentially expressed and differentially methylated (Up: up-regulated; Down: down-regulated; ND: no significant difference; Hypo: hypo-methylated; Hyper: hyper-methylated). **a):** number of differentially expressed REs that are hypo-methylated across all 12 cancer types; **b):** number of differentially expressed REs that are hyper-methylated across all 12 cancer types; **c):** number of differentially methylated REs that are up-regulated across all 12 cancer types; **d):** number of differentially methylated REs that are down-regulated across all 12 cancer types.
